# Community-Based Advanced Case Management for Patients with Complex Multimorbidity and High Medical Dependence: A Longitudinal Study

**DOI:** 10.3390/ijerph19137807

**Published:** 2022-06-25

**Authors:** Kana Kazawa, Michiko Moriyama

**Affiliations:** 1 Department of Medicine for Integrated Approach to Social Inclusion, Graduate School of Biomedical and Health Sciences, Hiroshima University, Hiroshima 734-8553, Japan; 2 Division of Nursing Science, Graduate School of Biomedical and Health Sciences, Hiroshima University, Hiroshima 734-8553, Japan; morimich@hiroshima-u.ac.jp

**Keywords:** advanced practice nurse, case management, disease management, chronic diseases, medical dependence, propensity score matching

## Abstract

This longitudinal study aimed to evaluate a community-based and nurse-led advanced case management model centered on disease management. Participants were chronically ill patients aged 20 years and older who were highly dependent on medical care. The case management group (CMG) received nurse-led advanced case management, and the comparison group (CG) was selected by matching estimated propensity scores with the CMG. We compared the changes in medico-economic indicators between the two groups and analyzed the physical and psychological indicators of the CMG over time. The CMG comprised 51 participants, of which eight dropped out by 12 months after registration. After 1:1 propensity score matching, there were 40 participants in the CMG and CG, respectively. At 12 months after the registration, there was no significant difference between the two groups and no change in the CMG. At 24 months after the registration, the CMG’s medical and long-term care costs decreased significantly, while the CG’s costs increased. Moreover, there was a significant reduction in the number of hospital days and hospital admissions in the CMG. Our findings revealed that nurse-led advanced case management could be useful for patients with complex needs to avoid hospitalization due to exacerbations.

## 1. Introduction

With the rise in the aging population, advancements in medical technology, and higher incidence of chronic diseases; it has become important to control the increasing costs of medical and long-term care, especially in developed countries [1]. Besides, medical and long-term care needs are becoming more complex and diverse due to the increase in the number of patients with chronic diseases and older patients with multiple conditions, in addition to changes in family structures such as a rise in the number of households comprising single people or older couples [2,3]. It has been reported that people with more complex needs often receive intermittent, uncoordinated, and ineffective services, leading to worsening health care dependency and increased cost incidence [4].

In this scenario, case management is a useful intervention methodology based on a comprehensive management and service coordination [5]. Case management is defined as “a collaborative process of assessment, planning, facilitation, care coordination, evaluation and advocacy for options and services to meet an individual’s and family’s comprehensive health needs through communication and available resources to promote patient safety, quality of care, and cost-effective outcomes [6]”. The efficacies of nurse-led case management focused on disease management for patients with high levels of medical dependence has already been reported [7,8,9,10,11]. A nurse-led case management program for hospital-discharged older adults with co-morbidities decreased hospital readmission rates and improved subjective health 12 months after the registration [7]. The intervention for patients with three or more emergency department visits, hospitalizations, or some combination thereof in the previous 12 months reduced the patients’ psychological distress six months after the registration [8]. A bibliographic review showed that nurse case management reduced the use of the emergency department, hospital admissions and medical costs, and improved quality of life (QOL) [9]. However, the evaluation period for these studies was approximately 12 months, so the long-term efficacies of nurse-led advanced case management are not clear. It is highly significant to verify whether the patients with medical dependence are stable for long periods of time.

In Japan, a society is aging, and medical and long-term care needs and costs continue to increase, while the working-age population responsible for care is decreasing. To ensure proper allocation and sustainable provision of care, the government is promoting a shift from “hospital-centered medical care” to an “integrated community care system.” This shift is marked by the integration of medical and long-term care so that residents can receive their medical and end-of-life care in their own communities [12]. In this care system we believe that providing community-based case management focusing on disease management jointly with medical insurers could be effective in improving the health outcomes of the patients with chronic diseases who are highly dependent on medical care, thereby controlling medical costs.

To achieve this, the case managers must have knowledge of medical, nursing, and long-term care. They should be able to provide support to the patient and the family in decision-making based on a comprehensive assessment of physical, mental, and social needs. Case managers should be able to construct preventive measures of disease management and coordinate care services with related parties. This approach is known as advanced case management.

Furthermore, in Japan, care managers are positioned in the long-term care insurance system to help older people formulate and coordinate care plans. Of these care managers, 66.9% have a basic qualification as a certified care worker or certified social worker without a medical background, 9.6% have a medical background, and only 3.3% are nurses [13]. Due to a low percentage of medically qualified care managers, it is difficult to take risk reduction measures to prevent disease. Furthermore, it is presumed that this current situation also disturbs effective information exchange and collaboration with medical professionals. We believe that trained nurses are best suited to play this role in advanced case management and that it is best to collaborate with care managers in the long-term care insurance system to manage patients who are highly dependent on medical care. Therefore, we developed a community-based and nurse-led advanced case management system, and the municipal government, a medical and long-term care insurer, placed trained nurses as candidates of an advanced practice nurse in the role of advanced case managers as the insurer’s project.

In this study, we aimed to evaluate the efficacy of advanced case management for 24 months. Based on earlier research [7,8,9,10,11], we hypothesized that case management focused on disease management will reduce chronically ill patients’ hospitalizations and emergency transports due to acute exacerbations, stabilize patients’ physical conditions, and ultimately reduce healthcare costs and improve quality of life (QOL).

## 2. Methods

### 2.1. Study Design

A longitudinal study using propensity score matching analysis was conducted which adhered to the Strengthening the Reporting of Observational Studies in Epidemiology (STROBE) guidelines [14]. The study period was from December 2015 to March 2019 and the nurse-led advanced case management was provided from 2015 to 2016.

### 2.2. Participants

The island area of Kure City was selected for this study. As of March 2016, the proportion of adults aged 65 and over in Kure City was 33.5%, and that in the island area was 59.7%, where many people aged 85 and over lived alone or as older couples.

#### 2.2.1. Participants in the Nurse-Led Advanced Case Management Group (Case Management Group; CMG)

The eligibility criteria were persons aged 20 years or older living on the island area of Kure City who were insured by the National Health Insurance (NHI) or the Late Elderly Health Insurance (LEHI) systems. The NHI includes persons who are ≤74 years old, individual business owners, those in the agriculture and fishing industries, short-time employees, and unemployed persons. LEHI covers persons aged 75 years or older as well as those who are 65 to 74 years old with specific impairments and other conditions. In our previous study, we identified the characteristics of chronically ill patients who were highly dependent on medical care using the health insurance claim data [4]. Based on this finding, we set the following criteria (see below), and the participants who met at least one of the conditions were identified as targets of the insurer’s project.

(1) Those whose most recent monthly medical expenses were one million yen or more.
(2) Those who had been hospitalized more than twice due to disease exacerbation in the last year.
(3) Those who had used the emergency transport in the last year.
(4) Those whose general condition was stable but who continued to be hospitalized because they were unable to find a place to recuperate.
(5) Those who visited the medical institutions for 10 days or more per month for the last three consecutive months or more.
(6) Those who had taken more than 10 kinds of oral medication for last three consecutive months.

The exclusion criteria were acute diseases and accidental injuries that did not require long-term treatment, as with other than chronic diseases. If the participant himself/herself was unable to make the decision, consent was obtained from their family members.

#### 2.2.2. Participants in the Comparison Group (CG)

The CMG in this study had a higher level of medical dependency, which meant a higher risk of death. In Japan, it has been reported that older people consume a larger proportion of medical services and incur greater costs before death [15]. Therefore, the CG comprised of individuals who were insured in Kure City as of April 2015 and could be followed for 12 months in order to compare the utilization and cost of medical services between the CMG and CG during the 12 months after registration, excluding deaths. After registering the CMG, we extracted the CG that was homogeneous with the CMG using propensity score matching from the health insurance claim data to reduce the selection bias.

Propensity scores were calculated by logistic regression analysis of receiving advanced case management regressed on age, sex, and medico-economic indicators during the 12 months before the registration (medical and long-term care costs, the number of hospitalization days, hospital admissions, outpatient visit days, and emergency transports, and use of advanced emergency medical care), the presence of heart failure (International Classification of Diseases 10th version Code; I50), stroke (I61, I63, I69), ischemic heart disease (I20–I25), and chronic kidney disease (N18), and long-term care level, using health insurance claims data. We performed 1:1 matching using the nearest-neighbor approach with replacement and a caliper width of 0.2 of the pooled standard deviation of the logit of the propensity score. We identified the presence of diseases associated with high medical dependence in previous studies [4].

**Figure 1:**
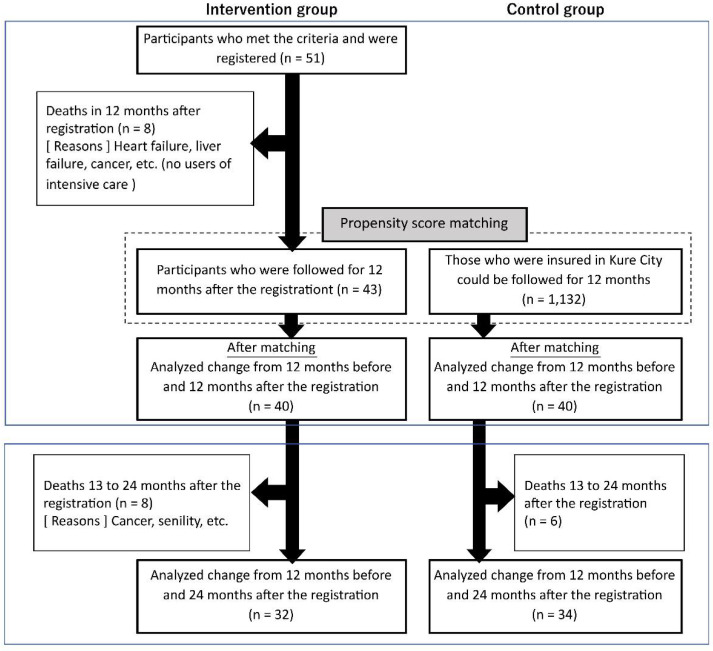
Flow chart of participant selection.

In this study, 51 patients who met the eligibility criteria were enrolled in the CMG, of which eight died within 12 months after registration. To compare the utilization and cost of medical services between the CMG and CG during the 12 months after registration, excluding deaths, we excluded those who died and selected 40 participants each in the CMG and CG by propensity score matching. After 24 months of registration, 32 and 34 patients in the CMG and CG were followed, respectively, excluding those who died (Figure 1). Although there were significant differences before propensity score matching (Table 1), there were no significantly different indicators in the baseline comparison between the two groups selected for matching (Table 2). Standardized differences were found for outpatient visit days and for the use of advanced emergency medical care, which were |0.13–0.15|, but all others were below |0.1| or less.

#### 2.2.3. Registration of the Participants

For the CMG, participants who met the eligibility criteria were selected from health insurance claim data or referrals from healthcare professionals, and their consent to participate in the study was obtained. For the CG, Kure City provided an opt-out on its public information website regarding the implementation of this joint research. Thereafter, the authors received the health insurance claim data from Kure City in a form that excluded personal information and used the data to register those who met the eligibility criteria.

### 2.3. Advanced Case Management

This study aimed to evaluate a community-based advanced case management model under the local public health insurance authority for Kure City. Based on the literature review, we defined case management operatively as follows: ”Nurses take the initiative in planning and adjusting care plans in multidisciplinary collaborations centered on disease management, daily life support, and psychological support, based on comprehensive assessment and decision support, for the stabilization of the physical and mental state and the maintenance and improvement of QOL of patients with chronic diseases who are highly dependent on medical care and have complex needs. Furthermore, to assure the quality of care, they manage in a timely and appropriate manner per the needs of patients through continuous monitoring and evaluation”. For the CMG, once the participants agreed to join it, the specially trained nurses provided the advanced case management. The period for providing advanced case management was around six months, with each initial face-to-face interview lasting approximately 60 min at the patient’s home, followed by repeated interviews and phone calls one to two weeks later, as needed.

First, an advanced case manager comprehensively assessed the patient’s current physical and mental conditions, symptoms, medical and long-term care needs, medical history, prescription medications, self-management adherence (diet, activities/exercise, medication/injection, alcohol and smoking), and environment (relationship with and supports by family, informal/formal care services and living environment). They also assessed if the patient’s need was compatible with the care services being used. Secondly, they shared the results of the assessment with the patient and their family and supported them in making decisions about where and how they wanted to receive care, including end-of-life care. The patient and family discussed advance care planning and documented this as needed. In this way, the unmet needs of the patient and family were identified, and case management was initiated to meet those needs. Third, the advanced case manager developed a care plan and coordinated multidisciplinary interventions such as medication, dietary therapy, and rehabilitation, as well as provided self-management education and asked family and home caregivers to assist with the patient’s activities of daily living (ADL). The patient and family implemented a self-management action plan to prevent disease exacerbations. Fourth, the advanced case manager provided psychological support to the patient and his/her family if they were anxious about recuperation and end of life.

### 2.4. Data Collection

As a pilot project, we set various outcomes. The major endpoints were medico-economic indicators (the number of days and times of hospitalization and the number of emergency transports caused by exacerbations, medical and long-term care costs, and outpatient visit days) of two groups within 12 months follow-up.

The other endpoints were as follows: the changes in the medico-economic indicators of the case management and control groups within 24 months and the physical (Barthel Index score) and psychological indicators such as the Euro QOL 5-dimensions 5-level (EQ-5D-5L) [16], and the Patient Health Questionnaire-9 (PHQ-9) [17] of the CMG within 12 months. The endpoints were collected at registration, and at 12 and 24 months after registration. Furthermore, as a qualitative evaluation of advanced case management, a questionnaire was administered to the participants and their families six months after registration.

### 2.5. Ethical Considerations

The study protocol was approved by the ethical committee of Hiroshima University. All procedures were carried out in accordance with the approved protocol and the Declaration of Helsinki and the Ethical Guidelines on Clinical Studies of Ministry of Health, Labour and Welfare. This study is registered under the following ID: UMIN000034440.

This study was conducted as the pilot project of Kure City in accordance with the personal information protection ordinance. The researchers developed advanced case management and supervised the project implemented as a medical insurer’s project by Kure City. Kure City removed the personal information from the data and provided it to us. We analyzed the anonymous data as an observational study.

### 2.6. Data Analysis

Medico-economic indicators were compared over time in terms of the amount of change during the 12 months before, 12 months after, and 13 to 24 months after registration. Physical and psychological indicators were compared at the times of six months after and 12 months after registration.

Normality was confirmed for each item and descriptive statistics were calculated. Chi-square tests, Freidman tests, Wilcoxon signed-rank tests, *t*-tests, and Mann-Whitney U tests were performed (where appropriate) using SPSS software version 25.0 (IBM, Armonk, NY, USA). The significance level was set at 5%.

## 3. Results

### 3.1. Changes from 12 Months before to 12 Months after Registration

Table 3 shows the results the comparison between groups and within groups from 12 months before to 12 months after registration. There were no differences between the groups. In the within-group comparison, the medical and long-term care costs and the number of outpatient visit days and emergency transports showed a decreasing trend in both groups, although there was no significant difference. Regarding the number of hospitalization days, there was a decreasing trend in the CMG and an increasing trend in the CG. Subsequently, the number of hospital admissions was unchanged in the CMG and significantly reduced in the CG (*p* = 0.026).

Table 4 shows the changes in the physical and psychological indicators of the CMG. Barthel Index and EQ-5D-5L did not change 12 months after registration. PHQ-9 increased slightly compared with the values at registration, however this change was not significant.

### 3.2. Changes from 12 Months before to 24 Months after Registration

Table 5 shows the results of comparison between and within groups from 12 months before to 24 months after registration. The results showed a significant decrease in medical and long-term care costs in the CMG (*p* = 0.021), whereas the CG showed a decrease after registration and an increase again after 13 months. (*p* = 0.048). Furthermore, the CMG showed a significant decrease in hospitalization days and number of hospital admissions (*p* = 0.002, *p* = 0.001, respectively). The CG showed a significant decrease in the number of hospital admissions after registration compared to 12 months before, but a slight increase after 13 months. The number of outpatient visit days and the frequency of emergency department use decreased after registration, although not significantly in both groups.

### 3.3. Evaluation of Case Management

**Figure 2:**
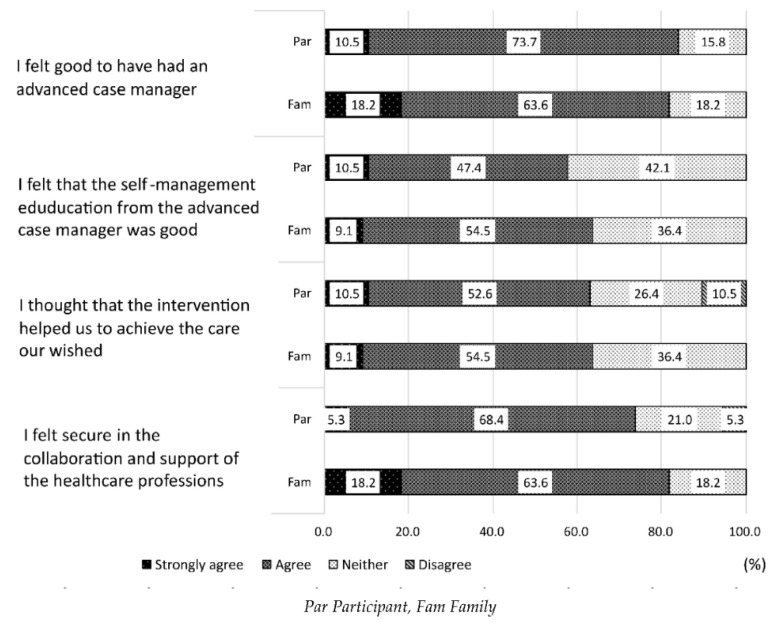
Evaluation of the case management by participants (*n* = 19) and family members (*n* = 11).

The evaluation of the advanced case management by the participants and their families is shown in Figure 2. The participants (84.2%) and 81.8% of the family members answered that they felt positive about having the advanced case managers, and 63.1% of the participants and 63.6% of the family members answered that the support enabled them to receive desirable medical and long-term care services. Furthermore, 73.7% of the participants and 81.8% of their family members felt relieved about the support provided by the healthcare professionals.

Since the participants were highly dependent on medical care, 16 of them died in the 24 months after registration. Of these, one died at home, and two were hospitalized with advanced emergency medical care in place at the time of death. The families of the participants who died said, “It was good to know what I didn’t know about aspiration pneumonia, home rehabilitation, and advance care planning. I could achieve the end-of-life care that my mother desired”, and that “It was good to know how to take care of my father with dementia. I think we were able to meet his wishes.”

## 4. Discussion

In this study, we evaluated if nurse-led advanced case management reduced the number of hospitalization days and the frequency of emergency department use and to maintain and improve the QOL of patients with chronic diseases who were highly dependent on medical care. It was assumed that the difference between the two groups would appear within 12 months of registration due to their high medical dependence. However, the results of the study showed no statistically significant change in the CMG at 12 months post-registration and no difference from the CG. Amazingly, in an analysis of changes at 24 months post-registration, however, the CMG’s medical and long-term care costs decreased significantly over time, while the CG’s costs decreased after registration and then increased again after 13 months. Furthermore, the CMG showed a significant decrease in the number of days and number of hospitalizations after registration. The difference in medical and long-term care needs between the two groups can be explained by the fact that almost all patients in both groups were hospitalized before registration and underwent surgery and intensive pharmacotherapy. Twelve months after registration, the conditions of both groups stabilized, and their medical and long-term care needs tended to improve. However, during that period, the CMG received subsequent self-management education and appropriate service adjustments to prevent disease and care deterioration. Therefore, it can be deduced that after 13 months of registration, the CMG was stable, while the CG may have required medical and long-term care due to exacerbations.

For the CMG, the advanced case managers first identified the medical and long-term care needs of the patients through a comprehensive assessment of the patients’ psychosocial aspects. We believe that this assessment, based on the knowledge and experience of the prognosis and the process of chronic diseases and the service coordination based on this assessment, contributed to the effective intervention [2,18,19]. For example, the advanced case manager collaborated with the physical therapist in cardiac rehabilitation in the case of patients with chronic heart failure and instructed the certified care workers with regard to activity strength and dietary therapy based on cardiac function and coping strategies during exacerbation of the patients. The advanced case manager educated patients at high risk for such things as diabetes and arranged clinic nurses to observe these conditions in the outpatient services. In these ways, even in cases where long-term care services had already been introduced, the advanced case manager added services that were insufficient and instructed preventive care methods to related care workers. We believe that this led to an improvement in the patient’s knowledge and motivation for disease management, as well as to a sense of security [20,21]. Additionally, many opportunities exist for advanced case managers to receive consultations from healthcare professionals on diseases and treatment, which is very important in continuous care for conducting needs assessments from multiple perspectives and sharing these with the multidisciplinary team [5,22,23].

Regarding decision-making support at the end of life, the families of patients who had died felt that the advanced case management helped them follow the patient’s wishes. Charles et al. state that shared decision-making is important in the patient-medical provider relationship when deciding on a treatment plan [24]. Shared decision-making emphasizes the importance of a process in which both providers and patients share information and try to agree on a desirable treatment choice, and the healthcare provider is required to support the patient’s proactive participation. In the cases we were involved in during this time, the patient’s wishes, anxiety about their worsening condition, and the family’s gratitude and anxiety toward the patient were expressed, and the case managers were able to confirm the patient’s important position in the family, educate the patient’s family about how to delay the worsening condition and respond to it early, to coordinate services, and to support the family’s anticipatory grief. Some patients who were not able to determine an end-of-life location were also satisfied with the advance care planning support provided by the case managers. By providing patients and their families with information, including their future outlook, we believe that it will be possible to approach the best process of end-of-life care for the patients themselves. Discussing end-of-life care and the related concerns of patients and their families also reduced their regrets and potential conflicts [25,26]. Therefore, it is very important to support patients’ decision-making and to consider best practices.

A limitation of this study is that the participants in this study were highly dependent on medical care, and many died. Therefore, it was difficult to determine the efficacy of the case management. We conducted a comprehensive evaluation that included objective indicators such as medico-economic and physical indicators, and subjective indicators, such as patient and family satisfaction with care and life and QOL. Further qualitative research will be needed to develop and refine the evaluation indicators.

## 5. Conclusions

This community-based and nurse-led advanced case management showed reduction of the number of hospitalization days and emergency department visits and maintenance and improvement of QOL of the patients with chronic diseases who were highly dependent on medical and long-term care. In this super aging society with complex healthcare needs, nursing care and care management should be more focused. We believe that allocating the advanced case managers in a community by the medical insurer and providing proactive case management are critically important for targeting insured persons with high medical dependency and controlling their healthcare costs and QOL through case management.

This system should be adopted as a policy at the national level and strategically introduced at the local government level. To achieve this goal, advanced case management education should be included in advanced practice nurse training and foster highly competent nurses. Our findings are beneficial for improving the quality of individual care for the patients with complex multimorbidity and high medical dependence, and for optimizing the allocation of the medical and long-term care service resources in the community.

In addition, patients with high medical dependence are at higher risk of death. Further research should be conducted to clarify whether community-based and nurse-led case management has contributed to patients’ advanced care planning including end of life planning.

## Figures and Tables

**Table 1:** Comparison of the two groups before propensity score matching. Mean ± Standard deviation.

Measures	Case Management Group (*n* = 43)			Comparison Group (*n* = 1132)			*p*-Value
Age (year)	83.2	±	6.5	80.0	±	9.2	0.027 *
Sex Male *n* (%)	16		(37.2)	446		(39.4)	0.874
Medico-economic indicators for 12 months before registration							
- Medical and long-term care costs (yen)	4,127,357.0	±	3,028,277.5	1,770,588.4	±	1,818,261.0	<0.001 ***
- Hospitalization days	75.8	±	63.9	32.5	±	77.8	<0.001 ***
- Number of hospital admissions	2.0	±	1.4	0.7	±	1.1	<0.001 ***
- Number of days of outpatient visits	31.4	±	36.1	48.0	±	49.7	0.001 **
- Number of emergency transports	0.9	±	1.1	0.4	±	0.8	<0.001 ***
- Use of advanced emergency medical care Yes *n* (%)	9		(20.9)	44		(3.9)	<0.001 ***
Level of long-term care required at registration *n* (%)							
- Requiring support level 1–2 (Low care level)	22		(51.2)	117		(10.3)	<0.001 ***
- Long-term care level 1–2 (Moderate care level)	8		(18.6)	134		(11.8)	
- Long-term care level 3–5 (Severe care level)	4		(9.3)	71		(6.3)	
- Not applicable ^§^	9		(20.9)	810		(71.6)	
Disease name at the registration (ICD-10 codes) Yes *n* (%)							
- Heart failure (I50)	29		(67.4)	484		(42.8)	0.002 **
- Ischemic heart disease (I20–I25)	22		(51.2)	395		(34.9)	0.034 *
- Stroke (I61, I63, I69)	18		(41.9)	502		(44.3)	0.876
- Chronic kidney disease (N18)	4		(9.3)	94		(8.3)	0.489

*ICD-10* International classification of diseases, 10th version. ^§^ Not applicable included those for whom information on their care level cannot be collected because they do not use long-term care services. *: *p* < 0.05, **: *p* < 0.01, ***: *p* < 0.001.

**Table 2:** Baseline comparison of the two groups after propensity score matching. Mean ± Standard deviation.

Measures	Case Management Group (*n* = 40)			Comparison Group (*n* = 40)			*p*-Value	Standardized Difference
Age (year)	83.5	±	6.2	83.9		10.1	0.831	−0.048
Sex Male *n* (%)	16		(40.0)	13		(32.5)	0.642	−0.079
Medico-economic indicators for 12 months before registration								
- Medical and long-term care costs (yen)	3,579,197.5	±	2,233,957.9	3,410,927.3	±	2,607,490.7	0.757	0.069
- Hospitalization days	70.8	±	63.4	64.0	±	84.2	0.684	0.091
- Number of hospital admissions	1.9	±	1.4	1.8	±	1.3	0.799	0.057
- Number of outpatient visits days	32.7	±	37.1	28.1	±	24.9	0.515	0.146
- Number of medical institutions used	2.8	±	1.3	2.8	±	1.6	1.000	0.000
- Number of emergency transports	0.9	±	1.1	0.8	±	1.4	0.727	0.078
- Use of advanced emergency medical care Yes *n* (%)	6		(15.0)	8		(20.0)	0.770	−0.132
Level of long-term care required at registration n (%)								
- Requiring help 1–2 (Low care level)	20		(50.0)	7		(17.5)	0.387	
- Long-term care level 1–2 (Moderate care level)	8		(20.0)	9		(22.5)		
- Long-term care level 3–5 (Severe care level)	4		(10.0)	6		(15.0)		
- Not applicable ^§^	8		(20.0)	18		(45.0)		
Disease name at the registration Yes *n* (%)								
- Heart failure (I50)	27		(67.5)	28		(70.0)	1.000	−0.054
- Ischemic heart disease (I20–I25)	19		(47.5)	21		(52.5)	0.823	−0.100
- Stroke (I61, I63, I69)	18		(47.5)	17		(42.5)	1.000	0.050
- Chronic kidney disease (N18)	4		(10.0)	5		(12.5)	1.000	−0.079

^§^ Not applicable included those for whom information on their care level cannot be collected because they do not use long-term care services.

**Table 3:** Change from 12 months before to 12 months after the registration.

			Case Management Group (*n* = 40), Comparison Group (*n* = 40)			
				Above: Mean, Below: (Standard Deviation)		
Medico-Economic Indicators		12 Months before Registration	12 Months after Registration	The Amount of Change	*p*-Value	
					Within Group ^a^	Inter-Group ^b^
Medical and long-term care costs (yen)	CMG	3,579,197.5 (2,233,957.9)	3,117,796.5 (2,409,920.8)	−461,401.0 (2,766,972.7)	0.104	0.597
	CG	3,410,927.3 (2,607,490.7)	2,972,947.8 (3,095,380.3)	−437,979.5 (1,652,810.8)	0.154	
Hospitalization days	CMG	70.8 (63.4)	66.7 (86.1)	−4.1 (89.6)	0.368	0.761
	CG	64.0 (84.2)	69.4 (112.0)	5.5 (65.9)	0.343	
Number of hospital admissions	CMG	1.9 (1.4)	1.8 (1.5)	−0.1 (1.8)	0.626	0.220
	CG	1.8 (1.3)	1.2 (1.5)	−0.6 (1.5)	0.026 *	
Number of outpatient visits days	CMG	32.7 (37.1)	31.2 (32.5)	−1.5 (17.0)	0.931	0.355
	CG	28.1 (24.9)	25.5 (27.7)	−2.5 (24.2)	0.139	
Number of emergency transports	CMG	0.9 (1.1)	0.5 (0.8)	−0.4 (1.2)	0.066	0.920
	CG	0.8 (1.4)	0.4 (0.9)	−0.4 (1.4)	0.065	

CMG case management group, *CG* comparison group. The amount of change = (total of 12 months after registration—total of 12 months before registration). ^a^: Wilcoxon test, ^b^: Mann-Whitney U test. *: *p* < 0.05.

**Table 4:** Changes in the physical and psychological indicators of the case management group. Mean ± standard deviation (*n* = 19).

	At the Registration			At 12 Months after the Registration			*p*-Value
Barthel Index	81.3	±	20.5	81.6	±	21.4	1.000
EQ-5D-5L	0.6098	±	0.2156	0.6566	±	0.1958	0.196
PHQ-9	3.9	±	4.1	5.1	±	5.4	0.754

The score range of the Barthel Index is from 0 to 100, with higher scores indicating the more independent the patient is in activities of daily living. EQ-5D-5L: the Euro QOL 5-dimentions 5- level. This score range is from 0 to 1, with higher scores indicating higher QOL. PHQ-9: Patient Health Questionnaire-9. This score range from 0 to 27, with higher total scores indicating higher levels of depression. Wilcoxon signed rank test.

**Table 5:** Changes from 12 months before to 24 months after the registration.

			Case Management Group (*n* = 32), Comparison Group (*n* = 34)		
			Above: Mean, Below: (Standard Deviation)		
		12 Months before Registration	12 Months after Registration	13 to 24 Months after Registration	*p*-Value
Medical and long-term care costs (yen)	CMG	3,359,299.4 (2,241,974.7)	2,812,704.1 (2,400,419.5)	2,749,320.6 (3,561,427.3)	0.021 *
	CG	3,272,133.5 (2,627,189.1)	2,904,419.1 (3,246,640.3)	3,651,219.4 (3,239,132.1)	0.048 *
Hospitalization days	CMG	61.5 (56.9)	53.7 (74.7)	25.7 (67.2)	0.002 **
	CG	60.0 (85.6)	63.9 (112.5)	71.0 (118.9)	0.111
Number of hospital admissions	CMG	1.5 (1.1)	1.5 (1.5)	0.8 (0.9)	0.001 **
	CG	1.7 (1.2)	1.0 (1.4)	1.2 (1.5)	0.017 *
Number outpatient visits days	CMG	31.7 (38.4)	29.4 (30.4)	25.7 (67.2)	0.928
	CG	28.4 (25.8)	24.9 (29.2)	24.9 (28.4)	0.054
Number of emergency transports	CMG	0.8 (1.1)	0.4 (0.8)	0.5 (0.8)	0.169
	CG	0.8 (1.5)	0.4 (1.0)	0.4 (1.1)	0.486

CMG case management group, CG comparison group. Friedman test. *: *p* < 0.05, **: *p* < 0.01.

## Data Availability

All data in this study received permission from Kure City as the insurer’s project. The datasets analyzed during the current study are not publicly available due the maintenance of confidentiality of our participants and declarations within the written information which participants had agreed on.
